# Strengthening local health research capacity in Africa: The African Doctoral Dissertation Research Fellowship Program

**DOI:** 10.11694/pamj.supp.2014.17.1.3729

**Published:** 2014-01-18

**Authors:** Caroline W Kabiru, Chimaraoke O Izugbara, Joyce Wairimu, Djesika Amendah, Alex C Ezeh

**Affiliations:** 1 African Population and Health Research Center, Manga Close off Kirawa Road, Kitisuru, P. O. Box 10787-00100, Nairobi, Kenya

**Keywords:** Health research, capacity building, fellowship program, Africa, ADDRF

## Introduction

Unlike many other regions of the world, sub-Saharan Africa still suffers massive deficits in doctorates. Yet, PhD training is key to building mastery of specific subject-matters and equipping researchers with the high-level skills needed to design, implement and evaluate development interventions and activities. Tettey [1] argues that the lack of PhDs in Africa is a major impediment to development in the continent. African universities which are expected to provide doctoral training face myriad challenges that impede their capacity to produce high quality PhD graduates to meet local development needs. These gaps in post-graduate training have resulted in numerous initiatives to enhance research capacity on the continent.

The African Population and Health Research Center (APHRC), a Nairobi-based regional research institute that conducts high-quality research on urbanization, population, health and education issues facing sub-Saharan Africa, has developed two complementary programs to address the research capacity gaps that plague the continent, particularly with respect to graduate training in population and public health as well as health systems research. The first, the Consortium for Advanced Research Training in Africa (CARTA) [2] brings together nine universities and four research institutions from West, East, Central, and Southern Africa and selected northern partners to build multidisciplinary research capacity in the broad fields of public and population health. CARTA offers a collaborative four-year doctoral training program in public and population health for junior faculty and researchers at participating African institutions. CARTA also has a strong institutional capacity building component that includes training workshops for faculty and staff and infrastructural investments. While CARTA addresses the important challenge of the scarcity of a robust research and training infrastructure capable of offering the type of vibrant and sustained doctoral training necessary to attract, train and retain the continent's brightest minds; the relatively small number of institutions involved in CARTA raises the urgent need for other innovative strategies to achieve greater reach in strengthening doctoral training in Africa. The second, the African Doctoral Dissertation Research Fellowship (ADDRF) Program, responds to this need and provides doctoral fellowships and targeted training workshops to facilitate rigorous research addressing governance, equity, health and population-related issues in Africa. The ADDRF Program provides a means to reach a wider number of PhD students from across the region (including both Anglophone and Francophone Africa) providing them the requisite guidance and transferable skills that will prepare them for academic and research positions. This supplemental issue of the Pan-African Medical Journal showcases some of the research conducted by fellows of the ADDRF Program.

## About the African Doctoral Dissertation Research Fellowship (ADDRF) Program

The ADDRF Program was initiated in 2008 by APHRC in partnership with the International Development Research Centre (IDRC). The Program aims to build a critical mass of the next generation of scholars committed to the reconstruction of the African academy and to facilitate rigorous research addressing health systems, governance, equity, and population-related issues in Africa. The Program achieves these objectives through the provision of PhD fellowships and targeted training workshops for fellows on critical research skills. Central to the ADDRF are methodology and advanced writing workshops designed to steadily enhance skills and knowledge, guide and propel the fellows through the research process, and provide a foundation for building networks of researchers, peers, and mentors. A detailed description of the rationale for and structure of the ADDRF Program is provided elsewhere [3]. The Program has supported 133 doctoral students from 20 Anglophone and Francophone sub-Saharan African countries. At the writing of this editorial, 61 (46%) of the fellows had completed their doctoral studies. The bulk of ADDRF graduates also continue to staff research and higher education institutions on the continent. Findings from a 2012 evaluation of the Program demonstrate several key successes in strengthening beneficiaries’ research capacity and enhancing retention. The evaluation covered 55 of 68 fellows in the first three cohorts who completed an electronic questionnaire. Out of these, 28 (51%) had graduated when the study took place. Results showed that 25 of the 28 graduates were working in their countries of origin; suggesting minimal ‘brain drain.’ Fellowships awarded to PhD students studying in sub-Saharan Africa-based universities—often in fellows’ countries of origin—can therefore be effective in reducing brain drain and enhancing retention. Almost all of the ADDRF graduates continue to work on health systems, governance, equity, and population-related issues and are making a contribution to knowledge production—22 out of the 28 graduates had publications in peer-reviewed journals within three years of receiving the award, several had undertaken policy-related consultancies since the completion of their studies, and 18 were involved in higher education teaching. Altogether, ADDRF fellows have published over 150 peer-reviewed papers in the last six years. Several of these papers are co-authored by more than one ADDRF fellow, sometimes from different countries. This is in tandem with ADDRF's commitment to promote scientific networking among Africans, break national barriers, and facilitate collaboration among fellows and across countries. Evaluation results also show that the training workshops have had an impact with about nine in ten fellows noting that the Program had improved their research methodology and scientific writing skills, as well as the quality of their dissertations. The evaluation also showed that the fellows felt that the Program had expanded their access to networking opportunities. Fellows largely rated the ADDRF Program's contribution to their educational experience in terms of access to networking opportunities/connections as excellent or very good. Other reported benefits of the Program included financial support for personal expenses, increased technical knowledge in one's field, improved communication skills, and personal development.

Managing the ADDRF Program over these years, we have realized the enormity of the need to support African doctoral students, an issue highlighted by other capacity building initiatives in the region [4]. For instance, the initial call attracted 118 applications from 19 countries in East, West, South, and Central Africa. However, less than a fifth of these were funded. In the first five years, over 750 applications were received, but less than one fifth of these were funded. Importantly, initial outcomes underscore the need for further investments to reach fellows from Francophone and Lusophone Africa. Since 2008, the number of applicants from Anglophone Africa has far exceeded the number from Francophone Africa. Currently, 17% (22 fellows) are from Francophone Africa. Over the years, the Program has made deliberate steps to reach potential applicants and to support fellows from Francophone Africa including wider dissemination of the call for application in French to research and academic institutions based in Francophone-Africa, the option to submit applications in French, the use of bilingual reviewers during selection, and the involvement of bilingual instructors in the training workshops.

ADDRF's initial vision of training and retaining a critical mass of scholars in the region remains as vital and relevant today as in 2008 when it was established. While several aspects of the African higher education environment are improving, the local production of quality PhDs continues to progress very slowly. Although universities in Africa face enormous constraints, they remain central to the creation of intellectual capital. Longer-term investment is thus needed to support and sustain the emergence of a critical mass of the next generation of scholars. The initial funding from IDRC has been critical in expanding the reach of the Program and has enabled the Center to leverage additional funding to support PhD training in the region. The additional funding has also expanded the scope of funded-research to areas beyond health systems research, such as sexual and reproductive health, giving APHRC the opportunity to leverage synergies between the ADDRF Program and its other research and research capacity strengthening programs, including CARTA. In the years ahead, the ADDRF will seek to sustain the momentum of its efforts to build a critical mass of the next generation of scholars as well as find innovative ways to secure the future of its graduates, primarily, by investing in their transition from PhD studentships to postdoctoral positions and beyond. In the long-run, however, the sustainability of the ADDRF and indeed, other research and training fellowship programs on the continent, will depend on investments from African governments and foundations.

## About the supplement

This supplement of the Pan-African Medical Journal showcases research conducted by 14 ADDRF fellows. The supplement is intended to promote fellows’ emergence as confident scholars and as internationally-recognized academics and researchers. Two papers by Saban and colleagues draw on nationally-representative data to examine the links between substance use and mental health among adolescents in South Africa. These papers draw attention to the mental health challenges faced by adolescents—an important, but often-understudied, aspect of health and wellbeing.

Sub-Saharan Africa has high levels of maternal and child morbidity and mortality [5]. Seven papers in this supplement are based on research on various aspects of maternal and child health (MCH). Ononokpono and Odimegwu draw on Demographic and Health Survey (DHS) data to describe the determinants of maternal health care utilization in Nigeria. Stephens and colleagues, examine the association between peripheral malaria parasitaemia and maternal anaemia and infant birthweight in Ghana. The paper on Barriers to antenatal syphilis screening in Burkina Faso examines barriers at facility- and community-levels that impede the uptake of antenatal syphilis screening. Echoka and colleagues report on the barriers that women face in accessing emergency obstetrics care services in a Kenyan coastal district with a large rural population. Kiondo and colleagues assess adverse neonatal outcomes in women with pre-eclampsia in Uganda. Two of the seven MCH-related articles highlight child health issues. The first paper, Anaemia among school children older than five years in the Volta Region of Ghana, shows that malaria parasitaemia, ferritin concentration and child's sex are associated with anemia in children. The second paper by Wado and colleagues investigates child vaccination status in rural Ethiopia and shows low overall levels of completion of child vaccination series. The authors also underscore the association between child vaccination status, parity and women's autonomy within the household. The authors call for interventions to enhance women's empowerment and access to family planning.

Although the majority of ADDRF fellows are engaged in health systems or public health research, the Program has also funded fellows conducting laboratory-based studies that have critical implications for health. For example, the paper by Ngure and colleagues examines the therapeutic properties of the East African greenheart (Canellaceae) in treating leishmaniasis, a neglected tropical disease currently treated through extensive hospitalization and the use of highly toxic and expensive drugs.

The remaining five papers report on various distal or proximate determinants of health. Okidu investigates HIV/AIDS communication in four Nigerian newspapers and argues that newspaper editors can do more to nurture an environment in which HIV/AIDS is discussed openly. Orem and colleagues, examine the role of research in informing health policy development in Uganda. Kretchy and colleagues assess adherence to anti-hypertensive medication among patients in two hospitals in Ghana and evaluate the extent to which an individual's locus of control is associated with adherence. Finally, two papers by Obembe and colleagues, and Mbada and colleagues report new and important research on the physical rehabilitation of patients with stroke and low back pain, respectively.

Overall, this collection of papers serves to demonstrate the wide range of topics that ADDRF fellows are researching. Each paper highlights programmatic and policy issues and it is our expectation that beyond showcasing the work of ADDRF fellows, this supplement will add to the body of research that can inform the formulation of programs and policies to improve health outcomes in sub-Saharan Africa.
